# Primary Care Considerations for Elderly U.S. Veterans of World War II and the Korean War: A Narrative Review

**DOI:** 10.7759/cureus.37309

**Published:** 2023-04-08

**Authors:** Joyce Akwe, Mary Ann Kirkconnell Hall

**Affiliations:** 1 Hospital Medicine, Atlanta Veterans Affairs Medical Center, Atlanta, USA; 2 Hospital Medicine, Emory University School of Medicine, Atlanta, USA

**Keywords:** toxic exposures, korean war, world war ii, veterans health, veterans

## Abstract

Many of the United States’ more than 18 million veterans obtain healthcare through the Department of Veterans Affairs’ (VA) Veterans Health Administration system; however, recent legislative changes have expanded veterans’ access to non-VA care in their communities, particularly for those who do not live near VA medical centers. Veterans are seen by physicians in outpatient practice across the United States and are admitted to non-VA hospitals; this is particularly salient for older veterans, who may require a more frequent and high level of care. We present a review of characteristics of U.S. veterans from two conflicts: World War II (WWII) and the Korean War. While non-VA clinicians are well equipped to provide care for patients of all different ages, veterans of armed conflicts have a unique constellation of exposures and cultural considerations that must be accounted for when providing them care. In this review, we describe characteristics of the generations of American veterans who served in WWII and the Korean War conflicts in a brief historical context. We then note conflict-specific exposures and potential long-term sequelae to watch for during physical examinations and to monitor thereafter, age-specific health and emotional concerns, and best practices for providing care to this cohort of veterans.

## Introduction and background

There were approximately 18.5 million veterans in the United States in 2021 [1]. While many receive care through the U.S. Department of Veterans Affairs’ (VA) Veterans Health Administration system and facilities, most get at least some medical care outside the VA health system. The VA MISSION Act, signed on June 6, 2018 [2], expanded veterans’ access to community-based care; the number of VA-enrolled veterans authorized for community care grew almost 70%, from 1.6 million in 2017 to 2.3 million, in 2020 [3].

Veterans of armed conflicts often have experiences and exposures that are different from non-veterans and that impact their health and function in the long term. Non-VA primary care clinicians are well equipped to care for patients of all different ages, including older adults with significant comorbidities. However, veterans' unique exposures (described in detail below along with sequelae to watch for) and cultural norms that must be considered when providing care have an additive effect on their health and quality of life. While most veterans appreciate the specialized care that is available through the VA, they are often able to access care more conveniently with less travel at non-VA community facilities.

Because of increases in non-VA healthcare utilization following enactment of the VA MISSION legislation [3], as well as the rapid aging and unique needs of these populations, we chose a narrative review methodology to provide useful guidance to primary care providers in a timely manner. We review characteristics of U.S. veterans from World War II (WWII) and the Korean War. As these groups age, the population of living veterans of each conflict will grow smaller, but their needs for health care will likely increase [4]. We provide brief historical descriptions, discuss concerns to note and monitor thereafter, and suggest best practices for providing care, based on literature, exposures, conflict culture, and age-specific health and emotional concerns.

World War II (1941-1945): the “GI” generation

There were an estimated 182,603 U.S. veterans of WWII as of 2021 [5]. Members of the "GI" generation (born between 1901 and 1927), so-called because they were the recipients of GI Bill educational benefits awarded by the U.S. government to returning WWII veterans, achieved an unprecedented level of educational achievement. Formal U.S. involvement in WWII lasted from December 7, 1941, when the Japanese bombed Pearl Harbor, until September 2, 1945. Across the Armed Forces, 16,112,566 Americans served, 291,557 died in battle, 113,842 died in non-combat settings, and 670,846 were wounded [6].

While most WWII veterans are men, women were instrumental in war efforts. Millions took wartime jobs, and more than 350,000 served in the Pacific, European, and Asian theaters, representing 2% of uniformed personnel. Though women were excluded from combat, many cared for men who were injured in battle as nurses. Women were not immune to wartime suffering: more than 400 lost their lives during the war.

Members of the GI generation tend to be team-oriented, patriotic, and attentive to rules [7].

The Korean War (1950-1953): the silent generation

As of 2021, there were approximately 800,000 U.S. veterans of the Korean War [1]. The Korean War began on June 25, 1950, and lasted until an armistice was signed on July 27, 1953. In all, 5,720,000 American men and women served in all the Armed Forces, 33,652 were killed in combat, 20,507 were killed outside battle settings, and 103,284 were wounded [6].

While a few “silent generation” (born 1925 to 1942) members fought in WWII, most were too young. Those who joined the military were much more likely to serve in the Korean War (and, to a lesser extent, the Vietnam War). These individuals faced numerous global adverse experiences in their childhoods, such as the Great Depression and WWII, in a context where “children should be seen and not heard” and authoritarian parenting approaches were dominant. Members of this generation often became “rebels without a cause,” starting adulthood as conformists who then began to challenge conventional culture. This generation produced America’s leading civil rights activists, its first “rock and roll stars,” antiwar leaders, and feminists.

## Review

Methods

To understand the needs of these special populations, we conducted a literature review using the National Library of Medicine’s PubMed database and an environmental scan of government materials (i.e., Centers for Disease Control and Prevention, VA) using Google searches. Our initial search identified 328 articles. We performed title and abstract review to determine which papers were relevant and reviewed PubMed’s “Similar Articles” and “Cited By” features of highly relevant papers, resulting in a set of 164 potentially relevant articles. We selected the 57 most salient resources for inclusion.

Unique health risks for WWII and Korean war veteran populations

Research has demonstrated that veterans of WWII and the Korean War have poorer self-rated health than non-veterans with similar sociodemographic characteristics [8]. Though WWII and the Korean War took place in separate areas of the world and with different styles of warfare, many exposures and subsequent health concerns are similar. Several innovations in healthcare took place between the wars, including widespread adoption of antibiotics and anti-tubercular agents.

All veterans should be assessed for exposure- and conflict-specific risks. Below, we detail areas of exposures, conflict-related risks, and related-health conditions, along with recommendations about how to solicit and utilize this information.

Physical Traumas

Though many decades have passed since any veterans incurred battle wounds during these conflicts, the effect of combat injuries - lost limbs (and traumatic amputations), head and brain injuries, gunshot and shrapnel wounds, and wounds that limit range of motion, among others [1,9] - can continue to impact daily life.

Dermatologic complaints were a primary cause for concern in the European theater, where 15%-45% of American sick call visits were for skin complaints [10]. Veterans who served in the Pacific theater or who were prisoners of war have elevated risks of different forms of skin cancer [11,12]. Besides wartime skin injuries, other health risks for WWII veterans include skin conditions caused by frostbite or cold injury; this is also a primary injury experienced by veterans of the Korean War, particularly those who fought in the Battle of the Chosin Reservoir (October-December 1950), where temperatures were extremely low for extended periods of time. Cold injuries can result in long-term changes in muscle, skin, nails, ligaments, and bones [13]. Limited evidence suggests an association between frostbite scars and non-melanoma skin cancers [14]. Neurologic complications (neuralgia, paresthesia in the extremities, sensations of hot or cold) and vascular injuries (e.g., Reynaud’s phenomenon) can also be long-term sequelae.

Practice recommendations: Ask the veteran about any war injuries that limit their functional abilities. Examples may include neuralgia, which can be well managed with medications such as gabapentin, or paresthesia/reduced sensation, which can raise the risk of falls, but can be countered with fall prevention measures. Perform a thorough evaluation of the skin, assessing for skin cancers and residual lesions from frostbite/cold injury. Assess for neurologic injury, focusing on symptoms such as bouts of pain in the extremities, and hot or cold and/or tingling sensations.

Affected veterans may need physical therapy to improve physical symptoms and/or occupation therapy consults to help them establish a safer home environment and task completion skills.

Psychological Injuries

Posttraumatic stress disorder (PTSD) develops after someone experiences or witnesses a life-threatening event. PTSD may present differently in older than younger veterans; the condition has been more extensively studied among veterans of the Vietnam War and later conflicts. Referred to as “combat fatigue,” “war neuroses,” or “shell shock” during WWII and the Korean War, PTSD was considered an unfortunate but unavoidable consequence of war. We thank the reviewer for this question and have amended the sentence to read: "Korean War veterans in particular are more likely to experience lifelong poorer mental health than veterans of other conflicts; this may be due to the greater prevalence of direct combat exposure or the closeness in time of this conflict to WWII, the largest conflict in human history” [15].

Studies of Vietnam and subsequent conflicts have demonstrated that veterans with PTSD have a higher risk of suicide, and those with comorbid PTSD and depression have higher risk than those with either condition alone [16,17]. PTSD is also associated with increased risk of cognitive decline and dementias [18], decreased bone mineral density [19], chronic back pain [20], and cardiovascular disease among veterans [21]. PTSD is strongly correlated with substance use disorder (SUD) [22]. SUD is more common among veterans than the general population, particularly among those with combat exposures, even decades later [23]. PTSD is also strongly associated with depression: when both conditions are present, the risk for metabolic syndrome is increased [24].

There is some evidence that PTSD can be exacerbated among older veterans by traumatic and stressful life experiences [25,26] and that PTSD from events in young adulthood can grow in severity in an individual’s later years, even after decades of fewer symptoms [27]. Traumatic brain injury has also been associated with an elevated lifetime risk of depression in men [28].

Veterans who were prisoners of war have unique vulnerabilities [19,29,30]. Their risk of PTSD is significantly higher in other veteran populations, and their experience is associated with an elevated risk of cardiovascular disease, including ischemic heart disease and hypertension [31]. WWII veterans who were prisoners of war can experience numerous social difficulties related to PTSD, including depression, and verbal and physical aggression in domestic relationships; the severity of these difficulties is directly correlated with the level of trauma they endured [32].

Practice recommendations: It is important to recognize when a veteran may be suffering from PTSD and refer them to mental health specialists who are trained in caring for patients with PTSD.

Assess if any veteran is experiencing symptoms of PTSD, which include flashbacks, nightmares, and severe anxiety, as well as uncontrollable thoughts about the event(s). PTSD symptoms may emerge years later. PTSD is more common after certain traumas, such as combat and sexual assault. Ask about recent exposures that could trigger PTSD symptoms.

Reassure veterans who are experiencing PTSD symptoms that there are many different treatment options available for PTSD, such as trauma-focused psychotherapy, including group and individual psychotherapy (in person and via telehealth) [33-35], mindfulness training [36], exposure therapies [37], medications, and combinations of these treatments. Treatments can often significantly improve sufferers’ quality of life and, in some instances, can eliminate symptoms altogether.

Assess veterans with PTSD for both depression and metabolic syndrome, as an association between the two has been demonstrated [24]. Monitor for exacerbations when they experience stressful or traumatic events. Ask veterans if they were prisoners of war and explain that war trauma is associated with PTSD and ongoing emotional challenges. Assist them in getting the care they need from a mental health care team that is trained and has experience with caring for veterans with PTSD, such as the nearest VA facility.

Exposure to Nuclear Materials

Between 1945 and 1962, the United States conducted atmospheric and underground nuclear tests in Pacific islands and in Nevada; service members during this time could be exposed before, during, and after such testing. Veterans were exposed to nuclear materials during the occupation of Hiroshima and Nagasaki, Japan (August 6, 1945, to July 1, 1946) following the United States’ detonation of two nuclear weapons, as were Americans who were prisoners of war in Japan in areas close to the two cities and who participated in the cleanup of contaminated areas.

The long-term impacts of radiation exposure are highly dependent on the dose received and its duration. While elevated rates of leukemia, solid cancers, and myelodysplastic syndromes (characterized by an increased risk of developing acute myeloid leukemia) have been observed in heavily exposed populations [38,39], the additional lifetime risk seems unlikely to be more than 30%-40% [40]; even heavy exposure is associated with <1 year of reduced life expectancy [40,41]. Cancer risk seems greater for individuals exposed at younger ages and rises over time [40,42]. Exposures received by aging veterans while they were young adults may thus result in a higher relative risk.

The VA recognizes a specific set of conditions as potentially associated with radiation exposure during military service, such as all forms of leukemia, except chronic lymphocytic leukemia; cancer of the thyroid, breast, pharynx, esophagus, stomach, small intestine, pancreas, bile ducts, gall bladder, salivary gland, urinary tract, brain, bone, lung, colon, or ovary; bronchioloalveolar carcinoma; multiple myeloma; lymphomas, other than Hodgkin’s disease; and primary liver cancer, except if there are indications of cirrhosis or hepatitis B [43]. Radiation exposure is associated with elevated risks of lens opacities/cataracts, thyroid problems, and cardiovascular diseases [41].

Practice recommendations: Ensure that patients have age-appropriate screenings for cancer, and ask about their radiation exposure and service history. Pay close attention to vision problems due to lens opacities, evaluate for cataracts if there are clinical concerns, and be mindful of the fact that veterans exposed to radiation can have an increased likelihood of thyroid problems and cardiovascular disease and consider screening them for thyroid and cardiovascular disease.

WWII Mustard Gas Exposures

Sulfur mustard (colloquially known as “mustard gas”) is a vesicant and blistering agent used during World War I as a chemical weapon [44,45]. During WWII, the U.S. Department of Defense recruited nearly 60,000 service members to “volunteer” for experiments using mustard agents to develop and assess countermeasures (clothing, ointments, and equipment) [45]. Levels of exposure varied widely, from small cutaneous patch tests to full-body exposures [45].

Mustard gas causes blistering to the skin, eyes, and lungs between 2 and 24 hours after contact [46]. Extensive skin burning following intense exposure (e.g., liquid form on skin) can be fatal. Eye exposure may cause blindness. Long-term effects of mustard gas inhalation include lung scarring and chronic respiratory disease (e.g., emphysema, chronic obstructive pulmonary disease) [44,45]. Causal relationships have been demonstrated between mustard gas exposure and lung/respiratory cancers, skin cancers, and leukemia, as well as psychological symptoms [44-46].

Practice recommendations: Assess veterans with this exposure (and others) for chronic respiratory disease and lung scarring. Consider screening for exposure-related cancers such as cancer of the lungs, nasopharynx, or larynx, or skin.

Noise

Hearing loss and tinnitus due to occupational noise damage/injury are prevalent among older adults and veterans, who were often exposed to loud noise during operations, training, and combat [9]. Hearing impairment can have significant impacts on daily living. Damaging noise, which was often unpredictable in occurrence and duration, could result from a number of military sources, including weapons, vehicles, sea vessels, aircraft, and even some communication devices, and/or activities common to both military and non-military settings, such as use of construction equipment or industrial machinery [47].

Practice recommendations: Evaluate the hearing of older adults and consider additional supports (such as using secure text messaging or resources such as TTY-based Telecommunications Relay Services) to establish and maintain connections with patients with hearing impairments. Additionally, make sure that their physical address is current; sensory deficits can make it more difficult to reach these individuals by telephone, and they may be more likely to miss appointments and follow-up.

Because individuals in this age group are more likely than younger people to have hearing impairments (whether service-related or not), it is important to minimize background noise and to always face the patient and speak clearly. The same is true for visual impairments; therefore, ensure that materials are available in large print versions as well.

Other Occupational Health Exposures

Both combat and non-combatant veterans may have been exposed to numerous dangerous substances [9]. Illnesses may result from occupational exposures to asbestos, lead, industrial solvents, and petroleum products. While there is evidence for long-term health impacts of some of these substances (e.g., exposure to asbestos is linked to asbestosis, pleural plaques, lung cancer, and mesothelioma) [48], it may be difficult to link them directly to sequelae that can be monitored. However, the VA has determined that veterans who served in several occupational areas were at elevated risk of harmful exposures, including carpentry and construction, demolition, manufacturing, milling, mining, shipyard work, and roofing and flooring installation [49].

Practice recommendations: Take a thorough occupational exposure history, especially for veterans who served in the above-mentioned professions. Asbestos-related diseases, such as shortness of breath, coughing, and chest pain, often do not appear until 20 to 50 years post-exposure.

Infectious Diseases

Despite the discovery of sulfa drugs and effective antibiotics before and during WWII, many American veterans suffered from infectious diseases. Nearly one million cases of tropical infections, mainly intestinal protozoa (mostly Entamoeba histolytica), helminths (hookworm infection), or malaria, were diagnosed during or shortly after WWII in U.S. troops, especially in the Pacific theater [50]. Dysentery and other diarrheal illnesses were also widespread [50].

At least 4,000 U.S. troops were infected with Plasmodium vivax during the Korean War, and many others were infected with Plasmodium falciparum [51,52]. Many were only diagnosed with malaria upon their return to the United States [53]. Splenomegaly was a common clinical finding; veterans who received splenectomies are subject to a number of long-term immunological sequelae, including reduced IgM concentrations, decreased antibody production, and defects in cellular immune function [54].

Practice recommendations: Veterans of the WWII Pacific theater and the Korean conflict may have experienced infections by capsulated organisms due to splenectomies. Patients without spleens may be more likely to experience overwhelming infections and greater mortality than individuals with functioning spleens. Keep this vulnerability in mind when patients have any signs of infection, particularly if they are unvaccinated for capsulated organisms.

Back to Today’s Encounter: Integrating Exposure Histories and Age-Specific Health and Emotional Concerns

All WWII veterans and most Korean War veterans are now age 80 or older [1,6], and many suffer from age-related ailments [4], including a higher risk of infection and dehydration, chronic illnesses, poor nutrition, and mobility issues including falls and gait instability. Look for any skin changes that may be concerning and assess their sensory abilities including handling of hot objects (especially water). Evaluate and discuss medication usage and related safety issues, nutrition, and exercise. Check (and monitor thereafter) personal hygiene, especially foot care [55].

Veterans in this age group are generally proud of their service to our country and enjoy talking about their years in the armed forces [4]. Asking them to share their military experiences and limiting any perception that they are being rushed to complete an encounter help them feel heard and respected for their sacrifice and service. When treating Korean War veterans, clinicians should remember this generation’s tendency to reject authority and embrace rebellion, and not take an authoritarian or patronizing approach.

During this stage of life, many may suffer from isolation as a result of loss of family members and friends, which has been associated with poorer overall health [56,57]. Evaluate for isolation-related conditions, such as depression, loneliness, and anxiety [57], and assess for sleep quality [58]. It is important not to assume that individuals in these age groups are incapable of retaining information, but do assess for and consider processing speed since older individuals may absorb and understand new information at a slower rate [59].

Practice recommendations: Track changes in address/residence and confirm that you can reach these patients after the encounter, as (1) frequent visits for ongoing concerns may necessitate close follow-up and (2) members of these populations may move into residential care or with family members for assistance. Many veterans in this age group tire easily; when providers need to instruct them on treatment or prognoses, it is best to teach while the veteran is at peak energy and to communicate information in short segments. Avoid rushing and allow additional time for making decisions and responding to questions.

## Conclusions

Every healthcare provider outside of the VA Healthcare system will likely eventually encounter a veteran patient, and it is important for physicians to be familiar with the challenges that veterans may face due to their service history and exposures. The service of American veterans of WWII and the Korean War should be recognized and honored, and their health and wellness protected as they proceed through their golden years.

## References

[1] (2023). United States Census Bureau. ACS 1-Year Estimates Subject Tables: American Community Survey: B21002 Period of military service for civilian veterans 18 years and over. https://data.census.gov/table?q=b21002&tid=ACSDT1Y2021.B21002.

[2] (2023). United States Senate Committee on Veterans' Affairs. The VA MISSION Act of 2018: The VA Maintaining Internal Systems and Strengthening Integrated Outside Networks (MISSON) Act [fact sheet]. https://www.veterans.senate.gov/services/files/E2D04FDE-4B08-4B84-988A-D5EEA907D1A7.

[3] Congressional Budget Office (2023). Congressional Budget Office. The Veterans Community Care Program: background and early effects. https://www.cbo.gov/publication/57583.

[4] Roefaro J (2021). The changing face of older American veterans. Sr Care Pharm.

[5] (2023). United States Census Bureau. Data from: American Community Survey: S2101 Veteran status. https://data.census.gov/table?q=s2101.

[6] (2023). Department of Veterans Affairs, Office of Public Affairs. America's wars. https://www.va.gov/opa/publications/factsheets/fs_americas_wars.pdf.

[7] Rickes PC (2016). Generations in flux. Plan Higher Educ.

[8] Fairman KA, Buckley K (2022). Association of potential for deaths of despair with age and military service era. Mil Med.

[9] (2022). US National Library of Medicine. Veterans and military health. https://medlineplus.gov/veteransandmilitaryhealth.html.

[10] Gelman AB, Norton SA, Valdes-Rodriguez R, Yosipovitch G (2015). A review of skin conditions in modern warfare and peacekeeping operations. Mil Med.

[11] Ramani ML, Bennett RG (1993). High prevalence of skin cancer in World War II servicemen stationed in the Pacific theater. J Am Acad Dermatol.

[12] Page WF, Whiteman D, Murphy M (2000). A comparison of melanoma mortality among WWII veterans of the Pacific and European theaters. Ann Epidemiol.

[13] Regli IB, Strapazzon G, Falla M, Oberhammer R, Brugger H (2021). Long-term sequelae of frostbite-a scoping review. Int J Environ Res Public Health.

[14] Imanbayev K, Makishev A, Zhagiparov M, McLoone P (2018). Non-melanoma skin cancers at sites of previous frostbite: case report and review. Case Rep Dermatol.

[15] Brooks MS, Fulton L (2010). Evidence of poorer life-course mental health outcomes among veterans of the Korean War cohort. Aging Ment Health.

[16] Bullman T, Schneiderman A, Gradus JL (2019). Relative importance of posttraumatic stress disorder and depression in predicting risk of suicide among a cohort of Vietnam veterans. Suicide Life Threat Behav.

[17] Nichter B, Norman S, Haller M, Pietrzak RH (2019). Psychological burden of PTSD, depression, and their comorbidity in the U.S. veteran population: suicidality, functioning, and service utilization. J Affect Disord.

[18] Yaffe K, Vittinghoff E, Lindquist K (2010). Posttraumatic stress disorder and risk of dementia among US veterans. Arch Gen Psychiatry.

[19] Hain RE, Hoyt RE, Moore JL, Linnville S, Segovia F, Ambrose MR (2011). Potential association of posttraumatic stress disorder and decreased bone mineral density in repatriated prisoners of war. Mil Med.

[20] Suri P, Boyko EJ, Smith NL (2019). Post-traumatic stress disorder symptoms are associated with incident chronic back pain: a longitudinal twin study of older male veterans. Spine (Phila Pa 1976).

[21] Beristianos MH, Yaffe K, Cohen B, Byers AL (2016). PTSD and risk of incident cardiovascular disease in aging veterans. Am J Geriatr Psychiatry.

[22] Jacobsen LK, Southwick SM, Kosten TR (2001). Substance use disorders in patients with posttraumatic stress disorder: a review of the literature. Am J Psychiatry.

[23] Branchey L, Davis W, Lieber CS (1984). Alcoholism in Vietnam and Korea veterans: a long term follow-up. Alcohol Clin Exp Res.

[24] Jakovljević M, Babić D, Crncević Z, Martinac M, Maslov B, Topić R (2008). Metabolic syndrome and depression in war veterans with post-traumatic stress disorder. Psychiatr Danub.

[25] Ogle CM, Rubin DC, Siegler IC (2014). Cumulative exposure to traumatic events in older adults. Aging Ment Health.

[26] Kaup BA, Ruskin PE, Nyman G (1994). Significant life events and PTSD in elderly World War II veterans. Am J Geriatr Psychiatry.

[27] Port CL, Engdahl B, Frazier P (2001). A longitudinal and retrospective study of PTSD among older prisoners of war. Am J Psychiatry.

[28] Kumar RG, Jayasinghe N, Walker RL (2021). Association of remote traumatic brain injury and military employment with late-life trajectories of depressive symptom severity. J Affect Disord.

[29] Hunt SC, Orsborn M, Checkoway H, Biggs ML, McFall M, Takaro TK (2008). Later life disability status following incarceration as a prisoner of war. Mil Med.

[30] Engdahl B, Dikel TN, Eberly R, Blank A Jr (1997). Posttraumatic stress disorder in a community group of former prisoners of war: a normative response to severe trauma. Am J Psychiatry.

[31] Kang HK, Bullman TA, Taylor JW (2006). Risk of selected cardiovascular diseases and posttraumatic stress disorder among former World War II prisoners of war. Ann Epidemiol.

[32] O'Donnell C, Cook JM, Thompson R, Riley K, Neria Y (2006). Verbal and physical aggression in World War II former prisoners of war: role of posttraumatic stress disorder and depression. J Trauma Stress.

[33] Steenkamp MM, Litz BT, Hoge CW, Marmar CR (2015). Psychotherapy for military-related PTSD: a review of randomized clinical trials. JAMA.

[34] Kitchiner NJ, Lewis C, Roberts NP, Bisson JI (2019). Active duty and ex-serving military personnel with post-traumatic stress disorder treated with psychological therapies: systematic review and meta-analysis. Eur J Psychotraumatol.

[35] Beech EH, Young S, Anderson JK, Belsher BE, Parr NJ (2022). Evidence-based Synthesis Program Reports. Evidence Brief: Safety and Effectiveness of Telehealth-delivered Mental Health Care. VA Evidence-based Synthesis Program Reports. Evidence Brief: Safety and Effectiveness of Telehealth-delivered Mental Health Care. Department of Veterans Affairs.

[36] Goldberg SB, Riordan KM, Sun S, Kearney DJ, Simpson TL (2020). Efficacy and acceptability of mindfulness-based interventions for military veterans: A systematic review and meta-analysis. J Psychosom Res.

[37] McLean CP, Levy HC, Miller ML, Tolin DF (2022). Exposure therapy for PTSD in military populations: a systematic review and meta-analysis of randomized clinical trials. J Anxiety Disord.

[38] Richardson D, Sugiyama H, Nishi N (2009). Ionizing radiation and leukemia mortality among Japanese Atomic Bomb Survivors, 1950-2000. Radiat Res.

[39] Iwanaga M, Hsu WL, Soda M (2011). Risk of myelodysplastic syndromes in people exposed to ionizing radiation: a retrospective cohort study of Nagasaki atomic bomb survivors. J Clin Oncol.

[40] Jordan BR (2016). The Hiroshima/Nagasaki survivor studies: discrepancies between results and general perception. Genetics.

[41] Douple EB, Mabuchi K, Cullings HM (2011). Long-term radiation-related health effects in a unique human population: lessons learned from the atomic bomb survivors of Hiroshima and Nagasaki. Disaster Med Public Health Prep.

[42] Preston DL, Ron E, Tokuoka S (2007). Solid cancer incidence in atomic bomb survivors: 1958-1998. Radiat Res.

[43] (2023). U.S. Department of Veterans Affairs, Veterans Benefits Administration. Presumptive disability benefits. Updated Ocobter.

[44] Hay A (1993). Effects on health of mustard gas. Nature.

[45] Institute of Medicine Committee on the Survey of the Health Effects of Mustard Gas and Lewisite (1993). Veterans at Risk: The Health Effects of Mustard Gas and Lewisite.

[46] (2023). Centers for Disease Control and Prevention. Facts about sulfur mustard. https://emergency.cdc.gov/agent/sulfurmustard/basics/facts.asp.

[47] Institute of Medicine (2006). Noise and Military Service: Implications for Hearing Loss and Tinnitus.

[48] (2022). Agency for Toxic Substances and Disease Registry. ToxFAQs for absestos. https://www.atsdr.cdc.gov/toxfaqs/tfacts61.pdf.

[49] (2022). Department of Veterans Affairs, Veterans Benefits Administration. Key changes to Veterans Benefits Manual M21-1, Part IV, “Compensation, DIC, and Death Compensation Benefits,” Subpart ii, “Compensation". https://www.benefits.va.gov/WARMS/docs/admin21/m21_1/mr/part4/subptii/ch02/9-24-15_Key%20Changes_M21-1IV_ii_2_SecF.docx.

[50] Mackie TT (1947). Tropical disease problems among veterans of World War II: preliminary report. Trans Am Clin Climatol Assoc.

[51] Beadle C, Hoffman SL (1993). History of malaria in the United States Naval Forces at war: World War I through the Vietnam conflict. Clin Infect Dis.

[52] Shanks GD (2022). Plasmodium vivax relapse rates in allied soldiers during the Second World War: importance of hypnozoite burden. Am J Trop Med Hyg.

[53] AQ JT, PA JA (1952). Malaria in returned veterans of the Korean war; clinical observations. J Am Med Assoc.

[54] Eibl M (1985). Immunological consequences of splenectomy. Prog Pediatr Surg.

[55] James K, Orkaby AR, Schwartz AW (2021). Foot examination for older adults. Am J Med.

[56] Donovan NJ, Blazer D (2020). Social isolation and loneliness in older adults: review and commentary of a national academies report. Am J Geriatr Psychiatry.

[57] Karel MJ, Wray LO, Adler G, Hannum AO, Luci K, Brady LA, McGuire MH (2022). Mental health needs of aging veterans: recent evidence and clinical recommendations. Clin Gerontol.

[58] Estes S, Tice JR (2021). Understanding and addressing the unique challenges and conditions of the veteran: improving sleep and well-being. Nurs Clin North Am.

[59] Spreng RN, Turner GR (2019). The shifting architecture of cognition and brain function in older adulthood. Perspect Psychol Sci.

